# FOXD3 is a novel tumor suppressor that affects growth, invasion, metastasis and angiogenesis of neuroblastoma

**DOI:** 10.18632/oncotarget.1579

**Published:** 2013-11-13

**Authors:** Dan Li, Hong Mei, Meng Qi, Dehua Yang, Xiang Zhao, Xuan Xiang, Jiarui Pu, Kai Huang, Liduan Zheng, Qiangsong Tong

**Affiliations:** ^1^ Department of Pediatric Surgery, Union Hospital of Tongji Medical College, Huazhong University of Science and Technology, Wuhan, P. R. China; ^2^ Clinical Center of Human Genomic Research, Union Hospital of Tongji Medical College, Huazhong University of Science and Technology, Wuhan, P. R. China; ^3^ Department of Cardiology, Union Hospital of Tongji Medical College, Huazhong University of Science and Technology, Wuhan, P. R. China; ^4^ Department of Pathology, Union Hospital of Tongji Medical College, Huazhong University of Science and Technology, Wuhan, P. R. China

**Keywords:** neuroblastoma, forkhead box D3, N-myc downstream regulated 1

## Abstract

The transcription factor forkhead box D3 (FOXD3) plays a crucial role in the development of neural crest cells. However, the function and underlying mechanisms of FOXD3 in the progression of neuroblastoma (NB), an embryonal tumor that is derived from the neural crest, still remain largely unknown. Here, we report that FOXD3 is an important oncosuppressor of NB tumorigenicity and aggressiveness. We found that FOXD3 was down-regulated in NB tissues and cell lines. Patients with high FOXD3 expression have greater survival probability. Over-expression or knockdown of FOXD3 responsively altered both the protein and mRNA levels of N-myc downstream regulated 1 (NDRG1) and its downstream genes, vascular endothelial growth factor and matrix metalloproteinase 9, in cultured NB cell lines SH-SY5Y and SK-N-SH. Luciferase reporter and chromatin immunoprecipitation assays indicated that FOXD3 directly targeted the binding site within NDRG1 promoter to facilitate its transcription. Ectopic expression of FOXD3 suppressed the growth, invasion, metastasis and angiogenesis of SH-SY5Y and SK-N-SH cells *in vitro* and *in vivo*. Conversely, knockdown of FOXD3 promoted the growth, migration, invasion and angiogenesis of NB cells. In addition, rescue experiments in FOXD3 over-expressed or silenced NB cells showed that restoration of NDRG1 expression prevented the tumor cells from FOXD3-mediated changes in these biological features. Our results indicate that FOXD3 exhibits tumor suppressive activity that affects the growth, aggressiveness and angiogenesis of NB through transcriptional regulation of NDRG1.

## INTRODUCTION

Neuroblastoma (NB), an embryonal tumor that is derived from the neural crest cells of sympathetic nervous system [1, 2], is the most common extracranial solid tumor in childhood, and accounts for approximately 7–10% of pediatric cancers and 15% of all pediatric cancer deaths [1, 3]. Although many factors that affect the tumorigenesis and metastasis of NB have been identified in recent years, such as signal transducer and activator of transcription 3 [4], Frizzled receptor 6 [5], and focal adhesion kinase [6], better elucidation of the mechanisms for the aggressive progression of NB is needed for improving the therapeutic efficiencies. Neural crest development is a complex and multi-step process, involving a series of inductive signals and responding transcription factors [7]. For example, the sonic hedgehog (Shh) signaling pathway is crucial for proper neural crest development through regulating the N-myc levels at both the transcriptional and posttranscriptional levels [8, 9]. Abnormal activation of Shh signaling pathway and amplification of N-myc have been demonstrated to participate in the development and progression of NB [1, 10], while inhibition of N-myc expression results in decreased survival of NB cells [11], suggesting that developmental factors may contribute to the tumorigenesis of NB. Thus, further investigation is warranted to identify the roles of neural crest development-related genes in the aggressiveness and progression of NB.

Forkhead box D3 (FOXD3), one member of the FOX transcription factor family, is originally identified in embryonic stem cells [12] and plays crucial roles in the neural crest development and stem cell biology through specifying the cell lineage [13-15]. In the early mouse embryo, FOXD3 is important for maintaining the pluripotent cells of inner cell mass [13], trophoblast progenitors [14], and neural crest [15], perhaps in part through regulation of Nanog and POU class 5 homeobox 1 [16]. Meanwhile, FOXD3 knockout results in early embryonic death in mice [13, 14]. Notably, FOXD3 is highly expressed during the wave of neural crest cell migration that forms peripheral neurons and glial cells [17–19], while FOXD3 silencing in early-migrating neural crest cells leads to an expansion of the melanoblasts [17]. These findings indicate that FOXD3 plays a primary role in neural crest development, and it is interesting to investigate the potential roles of FOXD3 in the neural crest-derived tumors.

Although previous studies have indicated that deregulation of certain FOX genes participates in the carcinogenesis [20], the functions and underlying mechanisms of FOXD3 in cancer still remain largely unknown. Recent evidence shows that ectopic expression of FOXD3 potently inhibits the growth of melanoma through inducing cell cycle arrest at G_1_ phase, which is associated with p53-dependent upregulation of p21^Cip1^ [21]. In gastric cancer specimens and cell lines, FOXD3 is under-expressed due to the promoter hypermethylation, and is correlated with survival time of patients with gastric cancer [22]. Over-expression of FOXD3 significantly inhibits the proliferation and invasion of gastric cancer cells *in vitro* and *in vivo*, at least partially, by promoting the apoptosis of cancer cells [22]. However, the exact function and downstream targets of FOXD3 in NB still remain elusive. In the current study, based on transcription factor binding site analysis, we predicted the FOXD3 as a regulator of N-myc downstream regulated 1 (NDRG1). We demonstrate, for the first time, that FOXD3 is down-regulated in NB tissues and cell lines, directly targets the NDRG1 promoter to facilitate its expression, and suppresses the growth, invasion, metastasis, and angiogenesis of NB cells *in vitro* and *in vivo*.

## RESULTS

### FOXD3 was under-expressed in NB tissues and cell lines

**Figure 1:**
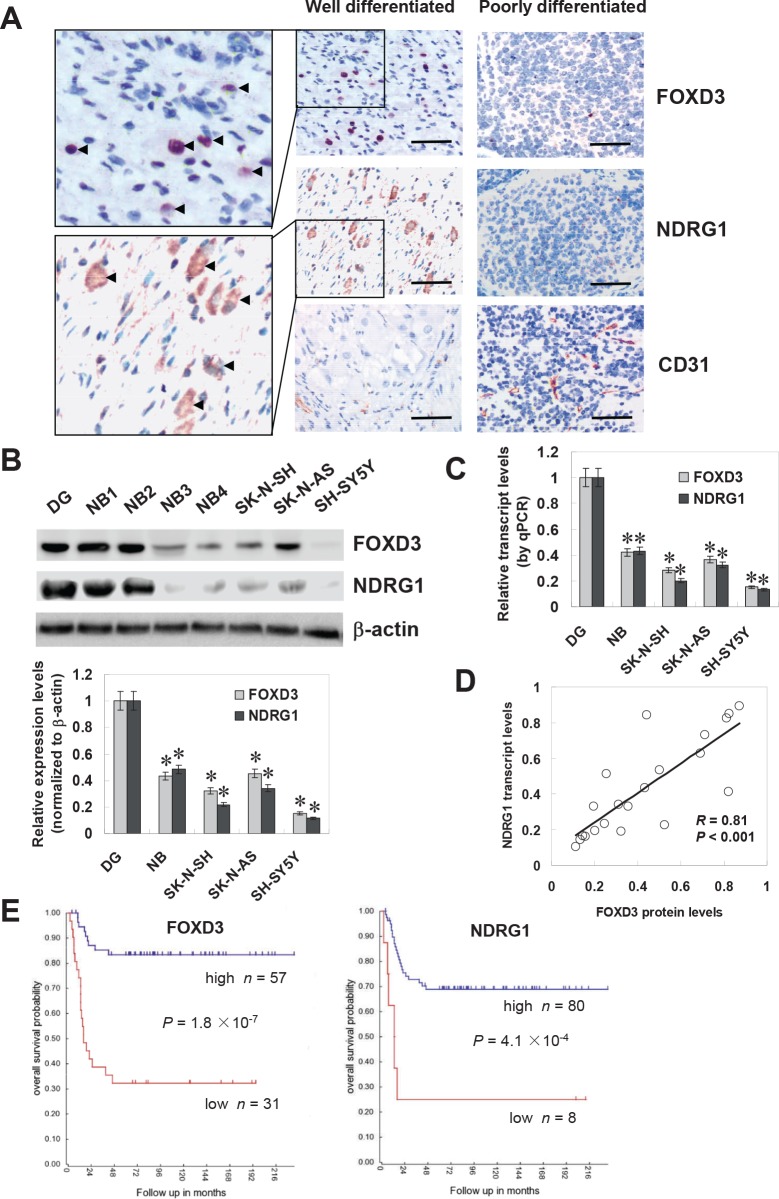
FOXD3 was under-expressed in NB tissues and cell lines **A**, immunohistochemical staining revealed that FOXD3 was expressed in the nuclei of tumor cells in NB specimens (arrowheads, brown). Cytoplasmic immunostaining of NDRG1 was noted in the tumor cells of NB specimens (arrowheads, brown). Scale bars: 100 μm. **B**, western blot indicated lower protein levels of FOXD3 and NDRG1 in NB tissues (n = 20) and cultured cell lines (SH-SY5Y, SK-N-AS, and SK-N-SH) than those in normal dorsal ganglia (DG; * *P* < 0.01 vs. DG). **C**, real-time quantitative RT-PCR revealed lower transcript levels of FOXD3 and NDRG1 in NB tissues (n = 20) and cultured cell lines (SH-SY5Y, SK-N-AS, and SK-N-SH) than those in DG (* *P* < 0.01 vs. DG). **D**, there was a positive correlation between FOXD3 protein and NDRG1 transcript levels in NB tissues (n = 20). **E**, Kaplan–Meier survival plots of 88 well-defined NB cases derived from R2 microarray analysis and visualization platform (http://r2.amc.nl) revealed that patients with high expression of FOXD3 or NDRG1 had greater survival probability than those with low expression.

Mining the publicly available clinical tumor expression data sets [R2: microarray analysis and visualization platform (http://r2.amc.nl)] revealed the altered FOXD3 transcript levels in some kinds of cancer, including down-regulation in colon cancer and cervix cancer, and up-regulation in renal cancer and endometrial cancer (Figure S1A), suggesting the potential roles of FOXD3 in tumorigenesis. In addition, FOXD3 transcript levels were lower in more aggressive NB tumors compared with the less aggressive neuroblastic tumors, ganglioneuroblastoma and ganglioneuroma (Figure S1B), and FOXD3 transcript levels were inversely associated with international neuroblastoma staging system (INSS) stages (Figure S1C). To further investigate the expression of FOXD3 in NB, paraffin-embedded sections from 42 well-established primary cases were collected [23]. Immunohistochemical staining revealed that FOXD3 was expressed in the nuclei of tumor cells (Figure 1A). FOXD3 expression was detected in 12/42 (28.6%) cases and the staining was weak in 6, moderate in 4, and intense in 2 (Table S1). The FOXD3 immunoreactivity was significantly higher in NB cases with good differentiation (*P* < 0.001), lower mitosis karyorrhexis index (MKI) (*P* = 0.003), and early INSS stages (*P* = 0.018) (Table S1). Notably, the immunostaining of NDRG1 (correlation coefficient *R* = 0.463, *P* = 0.002) and CD31 (correlation coefficient *R* = −0.411, *P* = 0.007) was associated with FOXD3 immunoreactivity in NB cases (Figure 1A and Table S2). The transcript levels of NDRG1 were also correlated with the aggressiveness of neuroblastic tumors (Figure S1B). Moreover, western blot and real-time quantitative RT-PCR were applied to measure the expression levels of FOXD3 and NDRG1 in subtotal 20 NB specimens, normal dorsal ganglia, and cultured SH-SY5Y, SK-N-AS, and SK-N-SH cell lines. As shown in Figure 1B and Figure 1C, lower protein and transcript levels of FOXD3 and NDRG1 were observed in NB tissues and cell lines than those in normal dorsal ganglia. There was a positive correlation between FOXD3 protein and NDRG1 transcript levels in NB tissues (correlation coefficient *R* = 0.81, *P* < 0.001, Figure 1D). Administration of DNA methyltransferase inhibitor 5-aza-2'-deoxycytidine (5-Aza-CdR) or pan histone deacetylase inhibitor trichostatin A (TSA) resulted in increased FOXD3 transcript levels in NB cells (Figure S2), indicating that epigenetic mechanisms were likely to be involved in the regulation of FOXD3. Kaplan–Meier survival plots of 88 well-defined NB cases derived from R2 microarray analysis and visualization platform revealed that patients with high FOXD3 (*P* = 1.8 × 10^−7^) or NDRG1 (*P* = 4.1 × 10^−4^) expression had greater survival probability than those with low expression (Figure 1E). These results indicated that FOXD3 was under-expressed and correlated with the expression of NDRG1 in NB tissues and cell lines.

### FOXD3 facilitated the expression of NDRG1 in cultured NB cell lines

**Figure 2:**
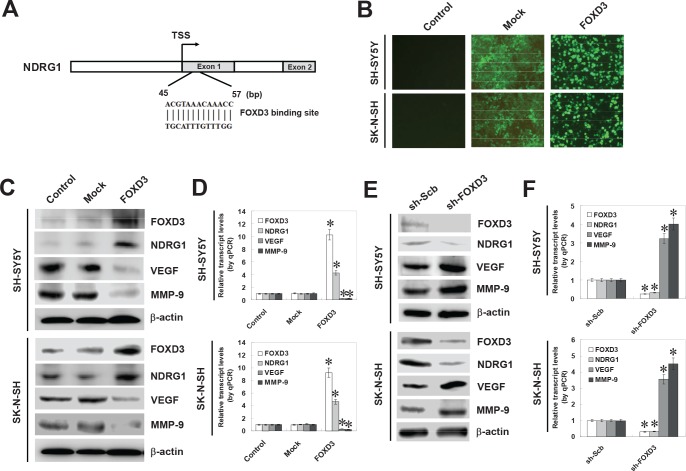
FOXD3 facilitated the expression of NDRG1 in cultured NB cell lines **A**, scheme of the potential binding site of FOXD3 within the NDRG1 promoter, locating at bases 45-57 downstream the transcription start site (TSS). **B**, transfection of FOXD3 into cultured SH-SY5Y and SK-N-SH cells resulted in nuclear expression of FOXD3 protein. **C** and **D**, western blot and real-time quantitative RT-PCR indicated that stable transfection of FOXD3 resulted in increased protein and transcript levels of FOXD3 and NDRG1 in SH-SY5Y and SK-N-SH cells, when compared to untransfected parental cells and those stably transfected with empty vector (mock). The expression levels of NDRG1 downstream genes, VEGF and MMP-9, were significantly down-regulated in FOXD3 over-expressing NB cells (* *P* < 0.01 vs. control). **E** and **F**, western blot and real-time quantitative RT-PCR indicated that stable transfection of short hairpin RNA targeting FOXD3 (sh-FOXD3) into SH-SY5Y and SK-N-SH cells, resulted in down-regulation of FOXD3 and NDRG1, and upregulation of VEGF and MMP-9, than those of scramble short hairpin RNA (sh-Scb)-transfected cells (* *P* < 0.01 vs. sh-Scb).

To investigate the hypothesis that FOXD3 may influence the expression of NDRG1 in NB, computational assessment was performed by transcription factor binding site analysis. In the NDRG1 promoter, one FOXD3 binding site was noted at bases 45-57 downstream the transcription start site (TSS) (Figure 2A). To explore the direct effects of FOXD3 on the expression of NDRG1 in NB cell lines, we performed the FOXD3 over-expression and knockdown experiments. Transfection of FOXD3 into SH-SY5Y and SK-N-SH cells resulted in nuclear expression of FOXD3 (Figure 2B). Western blot and real-time quantitative RT-PCR demonstrated that stable transfection of FOXD3 resulted in enhanced protein and transcript levels of FOXD3 and NDRG1 in NB cells, when compared to untransfected parental cells and those stably transfected with empty vector (mock) (Figure 2C and Figure 2D). In addition, the expression levels of NDRG1 downstream genes, vascular endothelial growth factor (VEGF) and matrix metalloproteinase 9 (MMP-9) [24], were significantly down-regulated in FOXD3 over-expressing NB cells (Figure 2C and Figure 2D). Since over-expression or knockdown of NDRG1 suppressed or promoted the expression of VEGF and MMP-9 in NB cells, respectively (Figure S3), and combining the evidence that there was no FOXD3 binding site within their promoters, we ruled out the possibility that FOXD3 might directly regulate the expression of VEGF or MMP-9. To further examine the suppressive role of FOXD3 on NDRG1 expression, we performed the FOXD3 knockdown experiments by stable transfection of short hairpin RNA (shRNA) targeting FOXD3 (sh-FOXD3) into SH-SY5Y and SK-N-SH cells. Transfection of sh-FOXD3 obviously down-regulated the expression of FOXD3 and NDRG1 (Figure 2E), and upregulated the protein levels of VEGF and MMP-9, than those of scramble short hairpin RNA (sh-Scb)-transfected cells (Figure 2E). Real-time quantitative RT-PCR analyses showed the down-regulated transcript levels of FOXD3 and NDRG1 and up-regulated transcript levels of VEGF and MMP-9 in NB cells transfected with sh-FOXD3, when compared with those transfected with sh-Scb (Figure 2F). In contrast, the transcript levels of several potential target genes bearing the FOXD3 binding sites within their promoters, including B-cell CLL/lymphoma 2 (BCL2), programmed cell death 4 (PDCD4), platelet derived growth factor C (PDGFC), and matrix metallopeptidase 14 (MMP-14), were not affected by stable over-expression or knockdown of FOXD3 in NB cells (Figure S4). Overall, these results demonstrated that FOXD3 considerably facilitated the NDRG1 expression at the transcriptional levels in NB cells.

### FOXD3 increased the transcription of NDRG1 through direct binding on its promoter

**Figure 3:**
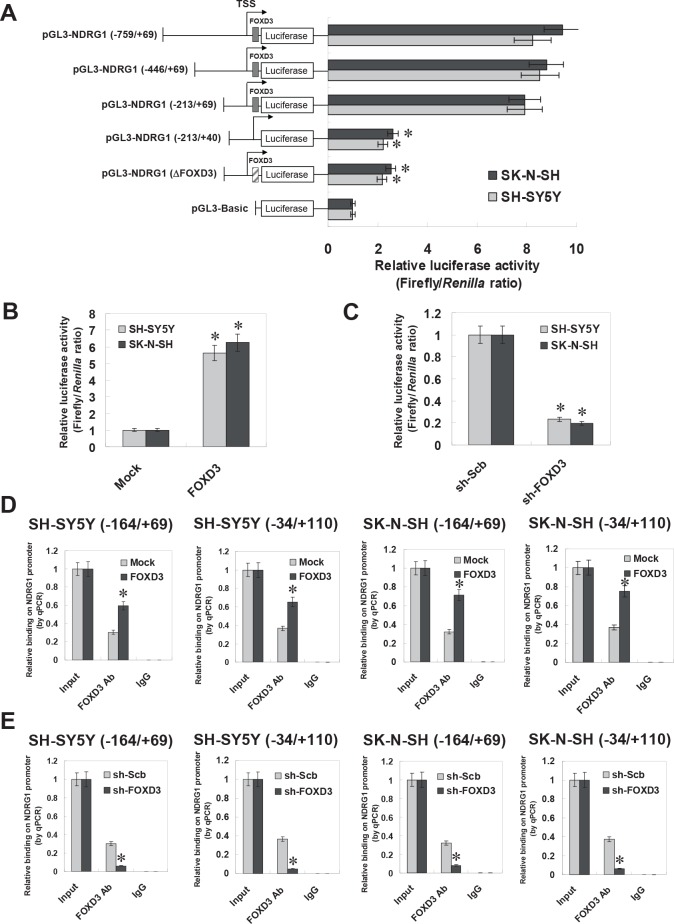
FOXD3 increased the transcription of NDRG1 through direct binding on its promoter **A**, dual-luciferase assay indicated that +41 to +69 bp relative to the transcription start site (TSS) was essential for the promoter activities of NDRG1, and mutation of FOXD3 binding site within this region resulted in decreased NDRG1 promoter activities in cultured SH-SY5Y and SK-N-SH cells (* *P* < 0.01 vs. pGL3-NDRG1−759/+69). **B**, stable transfection of FOXD3 resulted in increased luciferase activities of pGL3-NDRG1 (−759/+69) in SH-SY5Y and SK-N-SH cells, when compared with those stably transfected with empty vector (mock) (* *P* < 0.01 vs. mock). **C**, stable knockdown of FOXD3 resulted in decreased luciferase activities of pGL3-NDRG1 (−759/+69) in SH-SY5Y and SK-N-SH cells, when compared with those stably transfected with scramble short hairpin RNA (sh-Scb; * *P* < 0.01 vs. sh-Scb). **D**, chromatin immunoprecipitation (ChIP) and qPCR assay indicated that stable transfection of FOXD3 into NB cell lines SH-SY5Y and SK-N-SH, resulted in enrichment of FOXD3 on the −164/+69 and −34/+110 regions of NDRG1 promoter (* *P* < 0.01 vs. mock). **E**, ChIP and qPCR assay indicated that stable knockdown of FOXD3 with shRNA constructs decreased the binding of FOXD3 on the −164/+69 and −34/+110 regions of NDRG1 promoter in NB cells (* *P* < 0.01 vs. sh-Scb).

To determine whether FOXD3 could increase the transcription of NDRG1, a series of NDRG1 promoter fragments were generated, inserted into the pGL3-basic luciferase vector, and transfected into NB cells stably transfected with empty vector (mock) or FOXD3. Dual-luciferase assay indicated that +41/+69 bp relative to TSS was essential for the NDRG1 promoter activities, and mutation of FOXD3 binding site within this region resulted in decreased NDRG1 promoter activities in cultured SH-SY5Y and SK-N-SH cells (Figure 3A). Ectopic expression or knockdown of FOXD3 enhanced and abolished the promoter activities of NDRG1, respectively, in these NB cells (Figure 3B and Figure 3C). In addition, chromatin immunoprecipitation (ChIP) and quantitative PCR (qPCR) were applied to measure the enrichment of FOXD3 on NDRG1 promoter with two primer sets spanning its binding sites. Stable transfection of FOXD3 into NB cell lines SH-SY5Y and SK-N-SH resulted in enrichment of FOXD3 on the −164/+69 and −34/+110 regions of NDRG1 promoter (Figure 3D). Meanwhile, stable knockdown of FOXD3 with shRNA construct decreased the binding of FOXD3 on the −164/+69 and −34/+110 regions of NDRG1 promoter in NB cells (Figure 3E). These results indicated that FOXD3 directly interacted with the binding site within the NDRG1 promoter to increase its transcription.

### Ectopic expression of FOXD3 attenuated the growth, migration, invasion and angiogenesis of NB cells through targeting NDRG1

**Figure 4:**
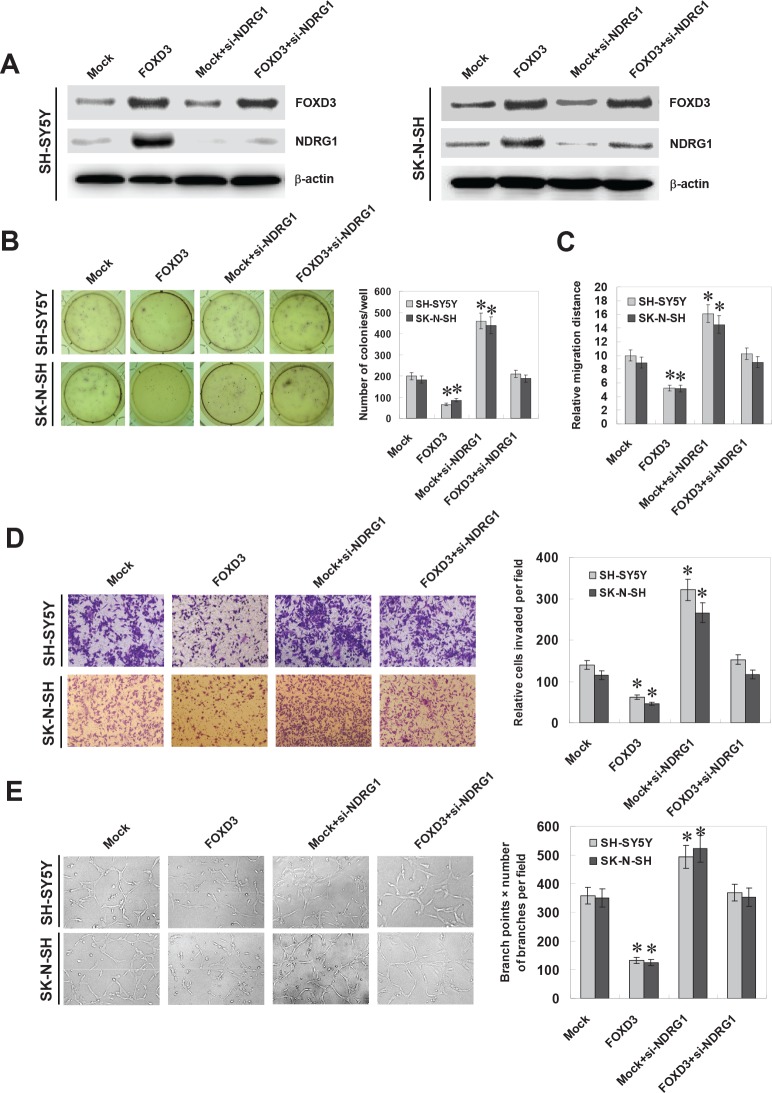
Ectopic expression of FOXD3 attenuated the growth, migration, invasion and angiogenesis of NB cells through targeting NDRG1 **A**, western blot indicated that stable transfection of FOXD3 expression vector into SH-SY5Y and SK-N-SH cells resulted in enhanced protein levels of FOXD3, when compared to those transfected with empty vetor (mock). Transfection of NDRG1 siRNA (100 nmol/L) restored the up-regulation of NDRG1 induced by stable FOXD3 over-expression. **B**, soft agar assay indicated the decreased colony formation capabilities of FOXD3 over-expressing SH-SY5Y and SK-N-SH cells than those of mock. Transfection of NDRG1 siRNA restored the colony formation capabilities of FOXD3 over-expressing cells (* *P* < 0.01 vs. mock). **C**, in scratch migration assay, the migration of FOXD3 over-expressing SH-SY5Y and SK-N-SH cells was significantly reduced when compared to mock. Transfection of NDRG1 siRNA rescued the migration of FOXD3 over-expressing cells (* *P* < 0.01 vs. mock). **D**, matrigel invasion assay indicated the decreased invasion capabilities of FOXD3 over-expressing SH-SY5Y and SK-N-SH cells than those of mock cells. However, transfection of NDRG1 siRNA restored the invasion of FOXD3 over-expressing cells (* *P* < 0.01 vs. mock). **E**, the tube formation of endothelial HUVEC cells was suppressed by treatment with the medium preconditioned by FOXD3 over-expressing SH-SY5Y and SK-N-SH cells, when compared to that of mock cells. Transfection of NDRG1 siRNA rescued the angiogenic capabilities of FOXD3 over-expressing cells (* *P* < 0.01 vs. mock).

Since previous studies indicate that NDRG1 participates in the growth, migration, invasion, and angiogenesis of cancer cells [24, 25], and combining the evidence that FOXD3 directly regulated the expression of NDRG1, we further investigated the effects of FOXD3 over-expression and NDRG1 restoration on cultured NB cells. Western blot and real-time quantitative RT-PCR indicated that knockdown of NDRG1 via small interfering RNA (siRNA) transfection resulted in its down-regulation and restored the FOXD3-induced upregulation of NDRG1 in SH-SY5Y and SK-N-SH cells (Figure 4A and Figure S5A). In soft agar assay, FOXD3 over-expression attenuated the anchorage-independent growth of SH-SY5Y and SK-N-SH cells, when compared to those stably transfected with empty vector (mock) (Figure 4B). On the other hand, restoration of NDRG1 expression with siRNA transfection rescued the NB cells from their defects in growth *in vitro* (Figure 4B). In scratch migration assay, FOXD3 over-expression attenuated the migration capabilities of SH-SY5Y and SK-N-SH cells (Figure 4C and Figure S5C). Transwell analysis showed that NB cells stably transfected with FOXD3 presented an impaired invasion capacity than mock cells (Figure 4D). The tube formation of endothelial cells was suppressed by treatment with the medium preconditioned by stable transfection of NB cells with FOXD3 (Figure 4E). In addition, restoration of NDRG1 expression via siRNA transfection rescued the SH-SY5Y and SK-N-SH cells from their defects in migration, invasion and angiogenesis induced by stable over-expression of FOXD3 (Figure 4C, Figure 4D, Figure 4E, and Figure S5C). However, over-expression of FOXD3 into cervix cancer HeLa cells did not affect the NDRG1 expression, although resulting in decreased *in vitro* growth and invasion capabilities (Figure S5E, Figure S5G, and Figure S5H). These results revealed the tumor suppressive roles of FOXD3 and indicated that upregulation of NDRG1 was involved in ectopic FOXD3 expression-inhibited aggressiveness and angiogenesis of NB cells.

### Knockdown of FOXD3 enhanced the growth, migration, invasion and angiogenesis of NB cells *in vitro*

**Figure 5:**
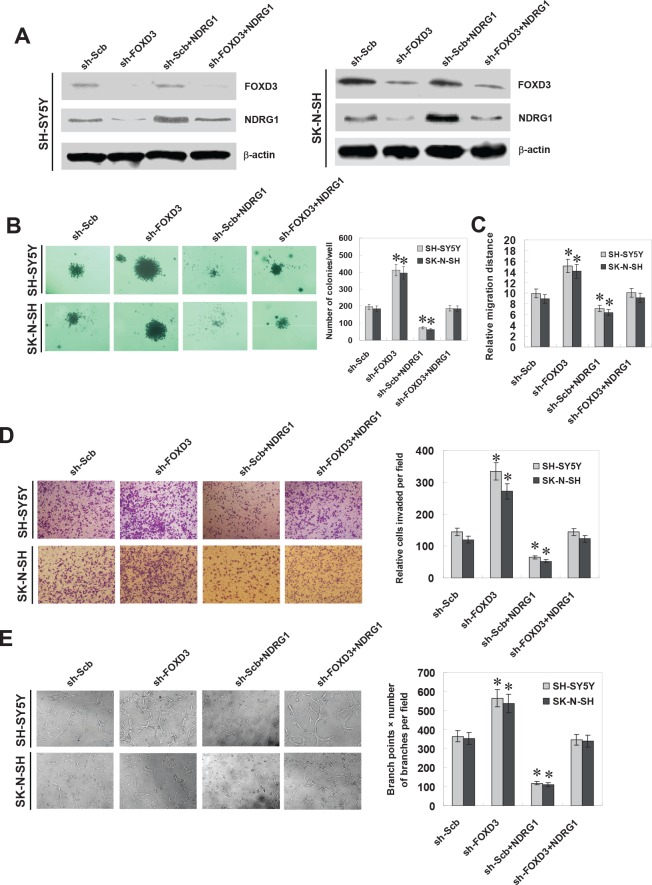
Knockdown of FOXD3 increased the growth, migration, invasion and angiogenesis of NB cells *in vitro* **A**, western blot indicated that stable transfection of short hairpin RNA targeting FOXD3 (sh-FOXD3) vector into SH-SY5Y and SK-N-SH cells resulted in decreased protein levels of FOXD3, when compared to those transfected with scramble short hairpin RNA (sh-Scb). Transfection of NDRG1 expression vector restored the down-regulation of NDRG1 induced by stable FOXD3 konckdown. **B**, soft agar assay indicated the enhanced colony formation capabilities of FOXD3 knocking down SH-SY5Y and SK-N-SH cells than those stably transfected with sh-Scb. Transfection of NDRG1 vector restored the colony formation of FOXD3 knocking down cells (* *P* < 0.01 vs. sh-Scb). **C**, in scratch migration assay, the migration of FOXD3 knocking down SH-SY5Y and SK-N-SH cells was significantly increased when compared to sh-Scb. Transfection of NDRG1 vector rescued the migration of FOXD3 knocking down cells (* *P* < 0.01 vs. sh-Scb). **D**, matrigel invasion assay indicated the increased invasion capabilities of FOXD3 knocking down SH-SY5Y and SK-N-SH cells than those of mock cells. However, transfection of NDRG1 vector restored the invasion of FOXD3 over-expressing cells (* *P* < 0.01 vs. sh-Scb). **E**, the tube formation of endothelial HUVEC cells was facilitated by treatment with the medium preconditioned by FOXD3 knocking down SH-SY5Y and SK-N-SH cells, when compared to that of mock cells. Transfection of NDRG1 vector rescued the angiogenic capabilities of FOXD3 knocking down cells (* *P* < 0.01 vs. sh-Scb).

To further explore the influence of FOXD3 on the aggressiveness and angiogenesis of NB cells, we further investigated the effects of FOXD3 knockdown and NDRG1 restoration on cultured NB cells. Western blot and real-time quantitative RT-PCR indicated that transfection of NDRG1 resulted in its over-expression and restored the down-regulation of NDRG1 induced by FOXD3 knockdown in SH-SY5Y and SK-N-SH cells (Figure 5A and Figure S5B). In soft agar assay, knockdown of FOXD3 facilitated the anchorage-independent growth of SH-SY5Y and SK-N-SH cells, when compared to those stably transfected with sh-Scb (Figure 5B). In migration assay, FOXD3 knockdown increased the migration capabilities of SH-SY5Y and SK-N-SH cells (Figure 5C and Figure S5D). Transwell analysis showed that NB cells stably transfected with sh-FOXD3 presented an increased invasion capacity than sh-Scb-transfected cells (Figure 5D). The tube formation of endothelial cells was increased by treatment with the medium preconditioned by stable transfection of NB cells with sh-FOXD3 (Figure 5E). In addition, restoration of NDRG1 expression via transfection of NDRG1 vector prevented the SH-SY5Y and SK-N-SH cells from their increase in growth, migration, invasion and angiogenesis induced by stable knockdown of FOXD3 (Figure 5B, Figure 5C, Figure 5D, Figure 5E, and Figure S5D). In contrast, knockdown of FOXD3 decreased the *in vitro* growth and invasion capabilities of renal cell carcinoma 786-O cells, without changes in the NDRG1 expression (Figure S5F, Figure S5G, and Figure S5H). These findings suggest that FOXD3 exhibits tumor-specific functions and identification of NDRG1 as a FOXD3 target gene may explain, at least in part, why FOXD3 suppressed the growth, invasion, metastasis and angiogenesis of NB cells.

### FOXD3 suppressed the growth, migration, invasion and angiogenesis of NB cells *in vivo*

**Figure 6:**
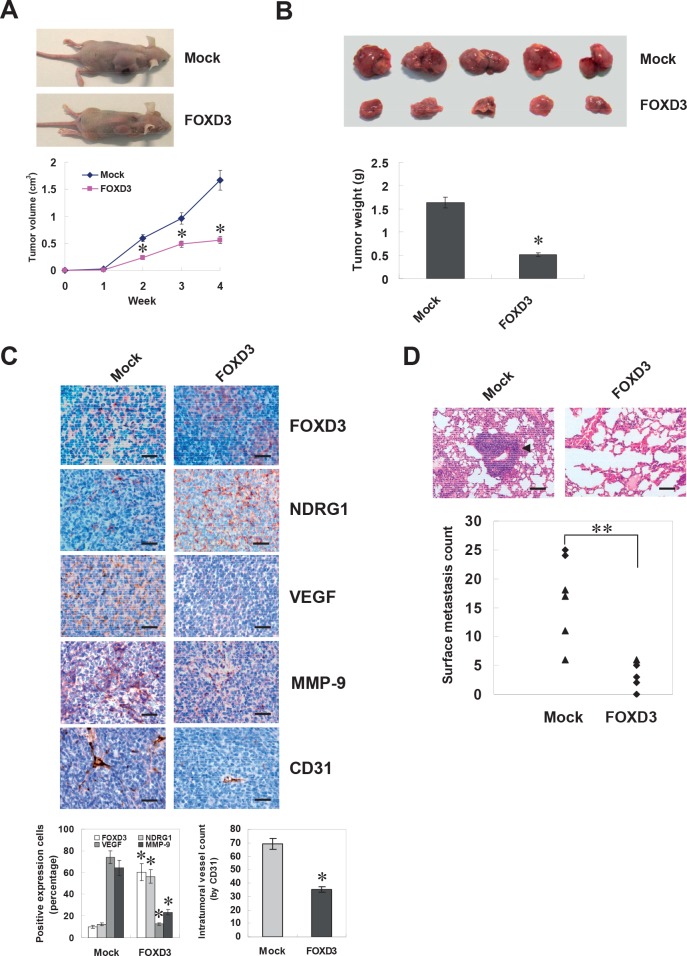
Over-expression of FOXD3 attenuated the growth, metastasis and angiogenesis of NB cells *in vivo* **A** and **B**, hypodermic injection of SH-SY5Y cells into athymic nude mice established subcutaneous xenograft tumors. Four weeks later, mice (n = 5) from each group were sacrificed. Stable transfection of FOXD3 into tumor cells resulted in decreased tumor size, when compared to those transfected with empty vetor (mock). The mean tumor weight formed from FOXD3 over-expressing cells was significantly decreased (* *P* < 0.01 vs. mock). **C**, immunohistochemical staining revealed that stable transfection of FOXD3 resulted in increased NDRG1 expression and decreased expression of VEGF and MMP-9 within tumors. The CD31-positive mean vessel density within tumors decreased after stable transfection of FOXD3 (* *P* < 0.01 vs. mock). Scale bars: 100 μm. **D**, SH-SY5Y cells were injected into the tail vein of athymic nude mice (n = 6 for each group). Tumor cells stably transfected with FOXD3 established significantly fewer lung metastatic colonies (arrowhead; ** *P* < 0.001 vs. mock). Scale bars: 100 μm.

We next investigated the efficacy of FOXD3 over-expression against tumor growth, metastasis and angiogenesis *in vivo*. Stable transfection of FOXD3 into SH-SY5Y cells resulted in decreased growth and tumor weight of subcutaneous xenograft tumors in athymic nude mice, when compared to those stably transfected with empty vector (mock) (Figure 6A and Figure 6B). In addition, the expression of FOXD3 and downstream genes NDRG1, VEGF, and MMP-9 was also altered by stable transfection of FOXD3 (Figure 6C). Moreover, stable transfection of FOXD3 resulted in a decrease in CD31-positive mean vessel density within tumors (Figure 6C). In the experimental metastasis studies, SH-SY5Y cells stably transfected with FOXD3 established statistically fewer lung metastatic colonies than mock group (Figure 6D). These results were consistent with the findings that over-expression of FOXD3 suppressed the growth, migration, invasion and angiogenesis of NB cells *in vitro*.

## DISCUSSION

Forkhead box (FOX) proteins, an evolutionarily conserved family of transcriptional regulators, mediate a wide spectrum of biological processes, such as metabolism, differentiation, proliferation, apoptosis, and migration [26, 27], and participate in the onset and progression of tumors [20]. Both gain- and loss-of-function studies have demonstrated that FOXD3 significantly inhibits the growth and invasion of gastric cancer cells [22]. In line with these findings, FOXD3 suppresses the migration and invasion of melanoma cells in a Rho-associated protein kinase dependent manner [28], and serves as a B-RAF target and novel cell cycle repressor in melanoma [21]. In the current study, we demonstrated the down-regulation of FOXD3 in NB tissues and cell lines. Since our preliminary data revealed that administration of DNA methyltransferase or histone deacetylase inhibitors resulted in enhanced FOXD3 levels in cultured NB cells, we hypothesize that the down-regulation of FOXD3 in NB may be due to aberrant promoter hypermethylation or histone acetylation, which warrants our further investigation. In addition, we found that ectopic expression of FOXD3 inhibited the growth, migration, invasion, and angiogenesis of NB cells, suggesting the tumor suppressive roles of FOXD3 during the progression of NB. Moreover, in publicly available clinical tumor expression data sets, we noted that FOXD3 was down-regulated in colon cancer and cervix cancer, while was up-regulated in renal cancer and endometrial cancer. Soft agar and transwell assays indicated that FOXD3 might exert tumor suppressive and oncogenic roles in cervix cancer and renal cancer, respectively. We believe that FOXD3 exhibits tissue-specific expression patterns and functions in human tumors, which warrants our further investigation.

As a member of the FOX family, FOXD3 is characterized by a monomeric DNA binding domain for nuclear localization and transcriptional regulation [12, 14, 16, 29]. Genome-wide location analysis has identified many proapoptotic genes as the potential transcriptional targets of FOXD3 in gastric cancer [22]. Ectopic FOXD3 expression potently inhibits melanoma cell growth and induces cell cycle arrest through upregulating the cyclin-dependent kinase inhibitor p21^Cip1^ in a p53-dependent manner [21]. In melanoma cells, FOXD3 down-regulates the cell migration and invasion through binding to the promoter of Rho family GTPase 3 [28]. FOXD3 can also indirectly prevent the binding of transcription factor paired box 3 on the promoter of microphthalmia-associated transcription factor [30]. Based on the transcription factor binding site analysis by Genomatix software (www.genomatix.de), we suspected several genes as the potential target of FOXD3, including NDRG1, BCL2, PDCD4, PDGFC, and MMP-14. However, our data revealed that FOXD3 did not transcriptionally regulate the expression of BCL2, PDCD4, PDGFC, or MMP-14 in NB cells. In this study, we found the positive correlation between FOXD3 and NDRG1 expression in NB specimens and cell lines. Importantly, restoration of NDRG1 expression rescued the NB cells from FOXD3-mediated suppressive phenotypes in growth, aggressiveness and angiogenesis, suggesting that FOXD3 may exert its tumor suppressive function, at least in part, through transcriptional regulation of NDRG1 in NB.

NDRG1, a 43 kD protein composed of 394 amino acids, was originally identified during the differentiation of colon carcinoma cell lines [31]. Subsequent studies have shown that NDRG1 exhibits tissue-specific expression patterns in human tumors. NDRG1 is down-regulated in colon cancer [32], prostate cancer [33], and glioma [34], while is up-regulated in breast cancer [35], liver cancer [36], and lung cancer [37]. A tumor suppressive role of NDRG1 has been reported so far. Previous studies have indicated a positive correlation between NDRG1 expression and patient survival, indicating that NDRG1 may be a prognostic biomarker in cancer patients [34]. In addition, NDRG1 inhibits the expression of VEGF and MMP-9, and suppresses the growth and angiogenesis of pancreatic cancer cells [24]. NDRG1 suppresses the metastasis of prostate cancer cells by inhibiting the activating transcription factor 3 [25]. Forced over-expression of NDRG1 into neuroepithelioma cell line results in reduced cell size and soft agar growth, implying the tumor suppressor role of NDRG1 in NB [38]. In this study, we demonstrated that NDRG1 was under-expressed in NB specimens and associated with patients' survival, and NDRG1 suppressed the growth, aggressiveness and angiogenesis of cultured NB cells, which is consistent with previous notion that NDRG1 is a potential therapeutic target of NB [39].

Recent evidence has shown that NDRG1 is inducible by a variety of factors and stimuli related to cancer progression, including oncogenes, tumor suppressors, and hypoxic microenvironment [40, 41]. In NB cells, NDRG1 expression is repressed by over-expression of N-myc [38]. Conversely, knockdown of N-myc in NB cells results in re-expression of NDRG1 [38], indicating that NDRG1 is one of the N-myc target genes. The von Hippel-Lindau tumor suppressor E3 ubiquitin protein ligase has also been shown to down-regulate NDRG1 expression in renal cancer cells by targeting hypoxia inducible factor 1 for degradation [42]. On the other hand, hypoxia induces NDRG1 expression through an early growth response 1 binding motif present in the NDRG1 promoter [43]. As a direct target gene of p53, NDRG1 is necessary for the execution of apoptotic program controlled by the tumor suppressor p53 [44]. Through a series of deletion of the promoter of NDRG1 luciferase constructs, we found that the +41 to +69 bp region was essential for the promoter activities of NDRG1 in NB cells, while mutation of the FOXD3 binding site within this region abrogated the promoter activities of NDRG1. In addition, ChIP assay indicated the enrichment of FOXD3 on the NDRG1 promoter. The direct role of FOXD3 in the positive regulation of NDRG1 gene was further evidenced by both over-expression and knockdown of FOXD3 in NB cells, suggesting that NDRG1 is a novel transcriptional target gene of FOXD3 in NB.

In summary, for the first time, we have demonstrated that FOXD3 is down-regulated in human NB, and FOXD3 exhibits tumor suppressor activity that affects the growth, invasion, metastasis, and angiogenesis of NB cells *in vitro* and *in vivo* through direct transcriptional regulation of NDRG1. This study extends our knowledge about the regulation of NDRG1 at the transcriptional level by tumor suppressive genes, and suggests that FOXD3 may be of potential values as a novel therapeutic target for NB.

## MATERIALS AND METHODS

### Patient tissue samples

Approval to conduct this study was obtained from the Institutional Review Board of Tongji Medical College. Paraffin-embedded specimens from 42 well-established primary NB cases were obtained from the Department of Pediatric Surgery, Union Hospital of Tongji Medical College, after the informed consent of the patients was obtained [23]. The pathological diagnosis of NB was confirmed by at least two pathologists. Based on Shimada classification system, including the MKI, degree of neuroblastic differentiation and stromal maturation, and patient's age, 19 patients were classified as favorable histology and 23 as unfavorable histology. According to INSS, 7 patients were classified as stage 1, 7 as stage 2, 9 as stage 3, 11 as stage 4, and 8 as stage 4S. In subtotal 20 NB patients, fresh tumor specimens were collected at surgery and stored at −80 °C until use. Protein and RNAs of normal human dorsal ganglia were obtained from Clontech (Mountain View, CA).

### Immunohistochemistry

Immunohistochemical staining was performed as previously described [23], with antibodies specific for FOXD3, NDRG1, VEGF, MMP-9 (Abcam Inc, Cambridge, MA; 1:200 dilutions), and CD31 (Santa Cruz Biotechnology, Santa Cruz, CA; 1:200 dilution). The reactivity degree was assessed by at least two pathologists without knowledge of the clinicopathological features of tumors. The degree of positivity was initially classified according to the percentage of positive tumor cells as the following: (−) < 5% cells positive, (1+) 6–25% cells positive, (2+) 26–50% cells positive, and (3+) > 50% cells positive. Slides with moderate positive (2+) or strong positive (3+) reactivity were classified as having a “high expression”, whereas slides with negative (−) or weak positive (1+) reactivity were classified as having a “low expression”.

### Western blot

Tissue or cellular protein was extracted with 1×cell lysis buffer (Promega, Madison, WI). Western blot was performed as previously described [45, 46], with antibodies specific for FOXD3, NDRG1, VEGF, MMP-9, and β-actin (Santa Cruz Biotechnology). Enhanced chemiluminescence substrate kit (Amersham, Piscataway, NJ) was used for the chemiluminscent detection of signals with autoradiography film (Amersham).

### Real-time quantitative RT-PCR

Total RNA was isolated with RNeasy Mini Kit (Qiagen Inc., Valencia, CA). The reverse transcription reactions were conducted with Transcriptor First Strand cDNA Synthesis Kit (Roche, Indianapolis, IN). The PCR primers for FOXD3 (NM_012183.2), NDRG1 (NM_001135242), VEGF (NM_001025366.2), MMP-9 (NM_004994.2), BCL2 (NM_000633), PDCD4 (NM_ 014456), PDGFC (NM_016205), MMP-14 (NM_004995), and β-actin (NM_001101.3) were indicated in Table S3. Real-time PCR was performed with SYBR Green PCR Master Mix (Applied Biosystems, Foster City, CA). The fluorescent signals were collected during extension phase, Ct values of the sample were calculated, and the transcript levels were analyzed by 2^−ΔΔCt^ method.

### Cell culture

Human NB cell lines SH-SY5Y (CRL-2266), SK-N-AS (CRL-2137) and SK-N-SH (HTB-11), cervix cancer cell line HeLa (CCL-2), renal cell carcinoma cell line 786-O (CRL-1932), and umbilical vein endothelial cells (HUVEC, CRL-1730) were purchased from American Type Culture Collection (Rockville, MD). Cells were grown in RPMI1640 medium (Life Technologies, Inc., Gaithersburg, MD) supplemented with 10% fetal bovine serum (Life Technologies, Inc.), penicillin (100 U/ml) and streptomycin (100 μg/ml), and applied for transcfection or treatment with 5-Aza-CdR (Sigma, St. Louis, MO) or TSA (Sigma) as previously described [47].

### Over-expression and knockdown of FOXD3

Human FOXD3 cDNA (1437 bp) was amplified from NB tissue, subcloned into the *Hind* III and *Xho* I restrictive sites of pEGFP-N1 (Clontech) and the *Hind* III and *BamH* I restrictive sites of pcDNA3.1 (Invitrogen, Carlsbad, CA), and validated by sequencing (Table S4). Seventy-two hrs post-transfection of pEGFP-N1 or pEGFP-FOXD3 vector, the localization of FOXD3 within tumor cells was monitored under a fluorescence microscope. The empty vector or pcDNA3.1-FOXD3 construct was transfection into tumor cells, and stable cell lines were screened by administration of neomycin (Invitrogen). The pcDNA3.1-transfected cells were applied as controls. The oligonucleotides encoding shRNA specific for FOXD3 and their scramble sequences were subcloned into the *Bam H* I and *Hind* III restrictive sites of GV102 (Genechem Co., Ltd, Shanghai, China; Table S4). The plasmids sh-FOXD3 and sh-Scb were verified by DNA sequencing and transfected into tumor cells with Genesilencer Transfection Reagent (Genlantis, San Diego, CA). Stable tumor cell lines transfected with shRNAs were screened by administration of neomycin (Invitrogen).

### Luciferase reporter assay

Human NDRG1 promoter (−759/+69 relative to TSS) and its truncation (−446/+69, −213/+69, and −213/+40) were amplified from genomic DNA by PCR (Table S4), inserted between the restrictive sites *Bgl* II and *Hind* III of firefly luciferase reporter vector pGL3-Basic (Promega), and validated by sequencing. The construct with a mutation of the FOXD3 binding site was generated with the mutagenic oligonucleotide primers (Table S4), according to the manual of GeneTailor Site-Directed Mutagenesis System (Invitrogen). Tumor cells were plated at 1×10^5^ cells/well on 24-well plates, and co-transfected with luciferase reporter vectors (30 ng) and *Renilla* luciferase reporter vector pRL-SV40 (10 ng, Promega). Twenty-four hrs post-transfection, firefly and *Renilla* luciferase activities were consecutively measured, according to the dual-luciferase assay manual (Promega). The firefly luciferase signal was normalized to the *Renilla* luciferase signal for each individual analysis.

### Target gene over-expression and knockdown

Human NDRG1 cDNA (1185 bp) was amplified from NB tissue (Table S4), subcloned into the *Hind* III and *Xho* I restrictive sites of pcDNA3.1/Zeo(+) (Invitrogen). To restore the FOXD3 knockdown-induced down-regulation of NDRG1, stable cell lines were transfected with the recombinant vector pcDNA3.1-NDRG1. To rescue the FOXD3-induced up-regulation of NDRG1, the 21-nucleotide siRNAs targeting the encoding region of NDRG1 were chemically synthesized (Genechem Co., Ltd) and transfected with Genesilencer Transfection Reagent (Genlantis). The scrambled siRNA (si-Scb) was applied as controls (Table S4).

### Chromatin immunoprecipitation

ChIP assay was performed according to the manufacture's instructions of EZ-ChIP kit (Upstate Biotechnology, Temacula, CA) [47]. DNA was sonicated into fragments of an average size of 200 bp. Different PCR primer sets were designed, distributing adjacent to the binding site of FOXD3 on NDRG1 promoter (Table S3). Real-time qPCR with SYBR Green PCR Master Mix was performed using ABI Prism 7700 Sequence Detector. The amount of immunoprecipitated DNA was calculated in reference to a standard curve and normalized to input DNA.

### Soft agar assay

Tumor cells at 5 × 10^3^ were mixed with 0.05% Nobel agar (Fisher Scientific, Pittsburgh, PA) in growth medium and plated onto 6-well plates containing a solidified bottom layer (0.1% Noble agar in growth medium). After the incubation of cells for 21 days, the number of cell colonies was counted under the microscope, and the cells were fixed with 100% methanol and stained with 0.5% crystal violet dye [45, 46].

### Scratch migration assay

Tumor cells were cultured in 24-well plates and scraped with the fine end of 1ml pipette tips (time 0). Plates were washed twice with phosphate buffer saline to remove detached cells, and incubated with the complete growth medium. Cell migration was photographed using 10 high-power fields, at 0 and 24 hrs post-induction of injury. Remodeling was measured as diminishing distance across the induced injury, normalized to the 0 hr control, and expressed as outgrowth (μm) [45, 48].

### Matrigel invasion assay

Matrigel invasion assay was performed using membranes coated with Matrigel matrix (BD Science, Sparks, MD). Homogeneous single cell suspensions (1 × 10^5^ cells/well) were added to the upper chambers and allowed to invade for 24 hrs at 37°C in a CO_2_ incubator. Invaded cells were stained with 0.1% crystal violet for 10 min at room temperature and examined by light microscopy. Quantification of invaded cells was performed according to published criteria [49, 50].

### Tube formation assay

Fifty microliters of growth factor-reduced matrigel were polymerized on 96-well plates. HUVECs were serum starved in RPMI1640 medium for 24 hrs, suspended in RPMI1640 medium preconditioned with tumor cells, added to the matrigel-coated wells at the density of 5×10^4^ cells/well, and incubated at 37°C for 18 hrs. Quantification of anti-angiogenic activity was calculated by measuring the length of tube walls formed between discrete endothelial cells in each well relative to the control [51, 52].

### *In vivo* growth, metastasis and angiogenesis assay

All animal experiments were approved by the Animal Care Committee of Tongji Medical College (approval number: Y20080290), and were conducted in accordance with national and international guidelines. For the *in vivo* tumor growth studies, 2-month-old male nude mice (n = 5 per group) were injected subcutaneously in the upper back with 1×10^6^ tumor cells stably transfected with empty or FOXD3 vectors. One month later, mice were sacrificed and examined for tumor weight, gene expression, and angiogenesis. The experimental metastasis (0.4×10^6^ tumor cells per mouse, n = 6 per group) studies were performed with 2-month-old male nude mice as previously described [45, 46].

### Statistical analysis

Unless otherwise stated, all data were shown as mean ± standard error of the mean (SEM). The SPSS 12.0 statistical software (SPSS Inc., Chicago, IL) was applied for statistical analysis. The χ^2^ analysis and Fisher exact probability analysis were applied for comparison among the expression of FOXD3, NDRG1, VEGF, MMP-9 and CD31, and individual clinicopathological features. Pearson's coefficient correlation was applied for analyzing the relationship between FOXD3 and NDRG1 expression levels. The Kaplan-Meier method was used to estimate survival rates, and the log-rank test was used to assess survival difference. Difference of tumor specimens or tumor cells was determined by *t* test or analysis of variance (ANOVA).

## Supplementary Figures and Tables


