# Supplementary material for: FOXD3 is a novel tumor suppressor that affects growth, invasion, metastasis and angiogenesis of neuroblastoma
Source: Oncotarget. 2013 Nov 13;4(11):2021–44. doi: 10.18632/oncotarget.1579 (PMC3875767; doi:10.18632/oncotarget.1579)
Supplement: Supplementary file 1 [file oncotarget-04-2021-s001.pdf]

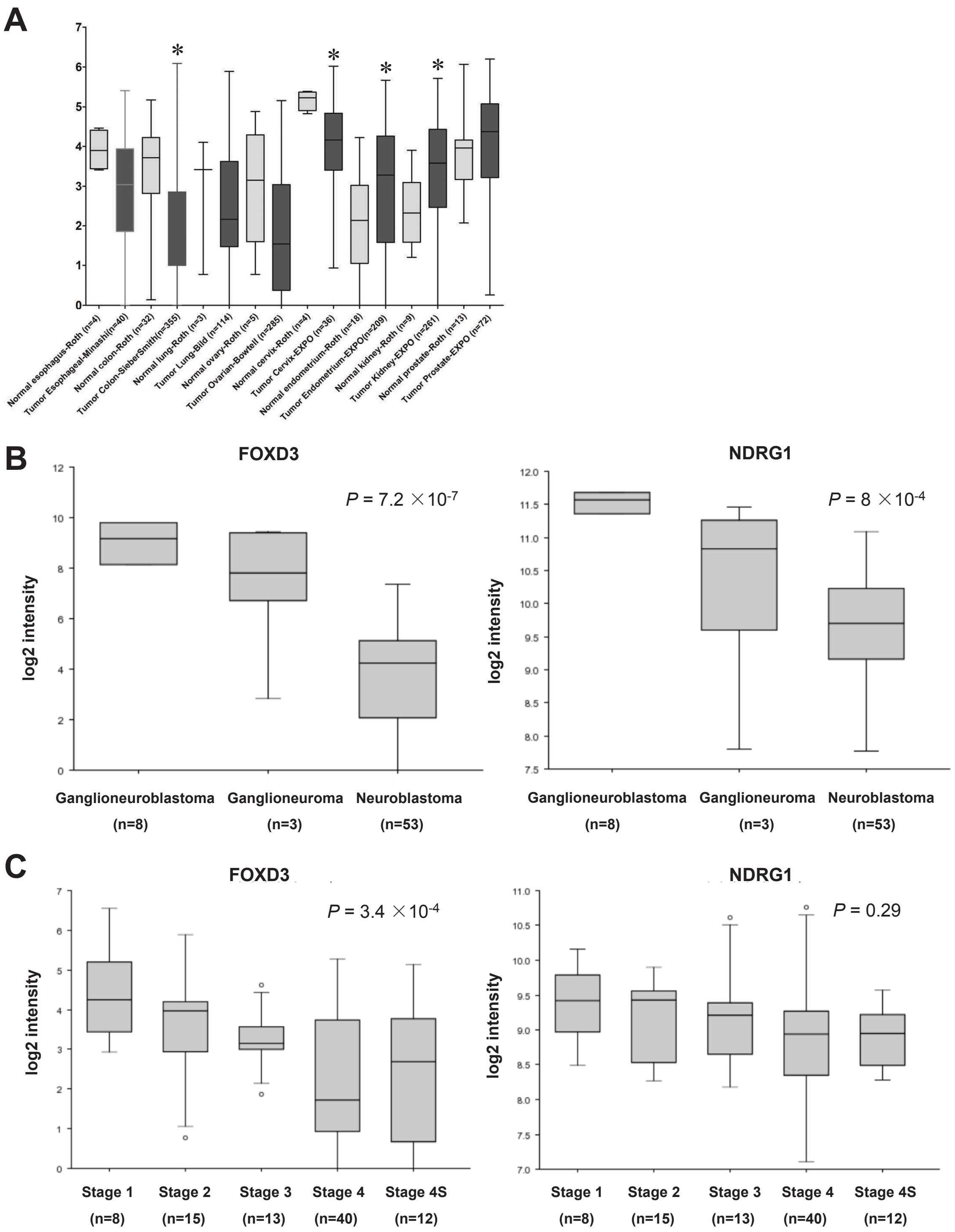

Supplementary Figure S1: Expression of FOXD3 and NDRG1 in publicly available clinical tumor data sets. (**A**) mining the publicly available clinical tumor expression data sets [R2: microarray analysis and visualization platform (http://r2.amc.nl)] revealed that FOXD3 was significantly down-regulated in colon cancer and cervix cancer, while was up-regulated in renal cancer and endometrial cancer (* $P < 0.05$ vs. corresponding normal tissues). (**B**) the microarray expression data derived from 64 well-defined neuroblastic tumors (R2: microarray analysis and visualization platform) revealed that FOXD3 and NDRG1 transcript levels were lower in more aggressive NB tumors compared with the less aggressive ganglioneuroblastoma and ganglioneuroma. (**C**) the microarray expression data derived from 88 well-defined NB specimens (R2: microarray analysis and visualization platform) indicated that FOXD3 transcript levels were inversely associated with INSS stages.

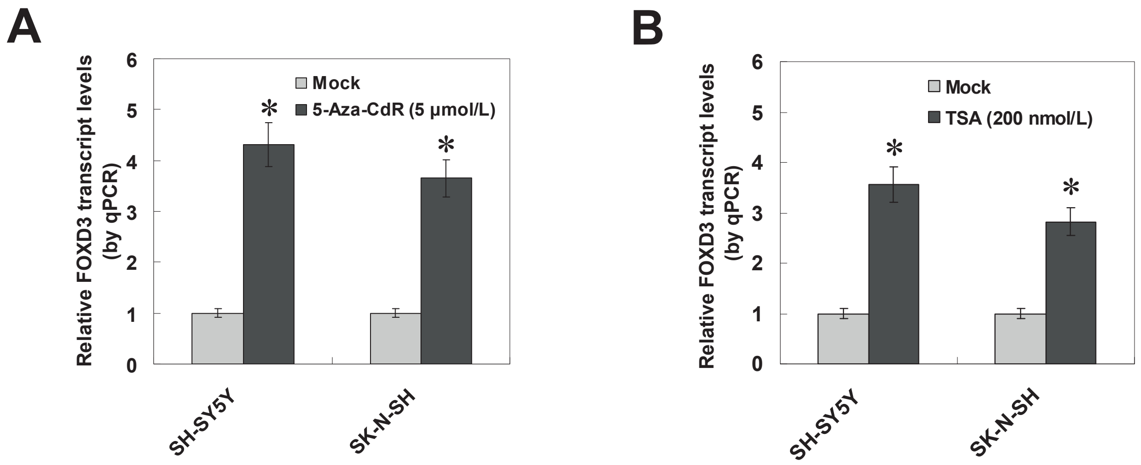

Supplementary Figure S2: Epigenetic regulation of FOXD3 in NB cells. Real-time quantitative RT-PCR indicated that administration of DNA methyltransferase inhibitor 5-aza-2'-deoxycytidine (5-Aza-CdR, 5 μmol/L) or pan histone deacetylase inhibitor trichostatin A (TSA, 200 nmol/L) for 24 hours resulted in increased FOXD3 transcript levels in NB cells, when compared to those treated with solvent (mock; * $P < 0.01$ vs. mock).

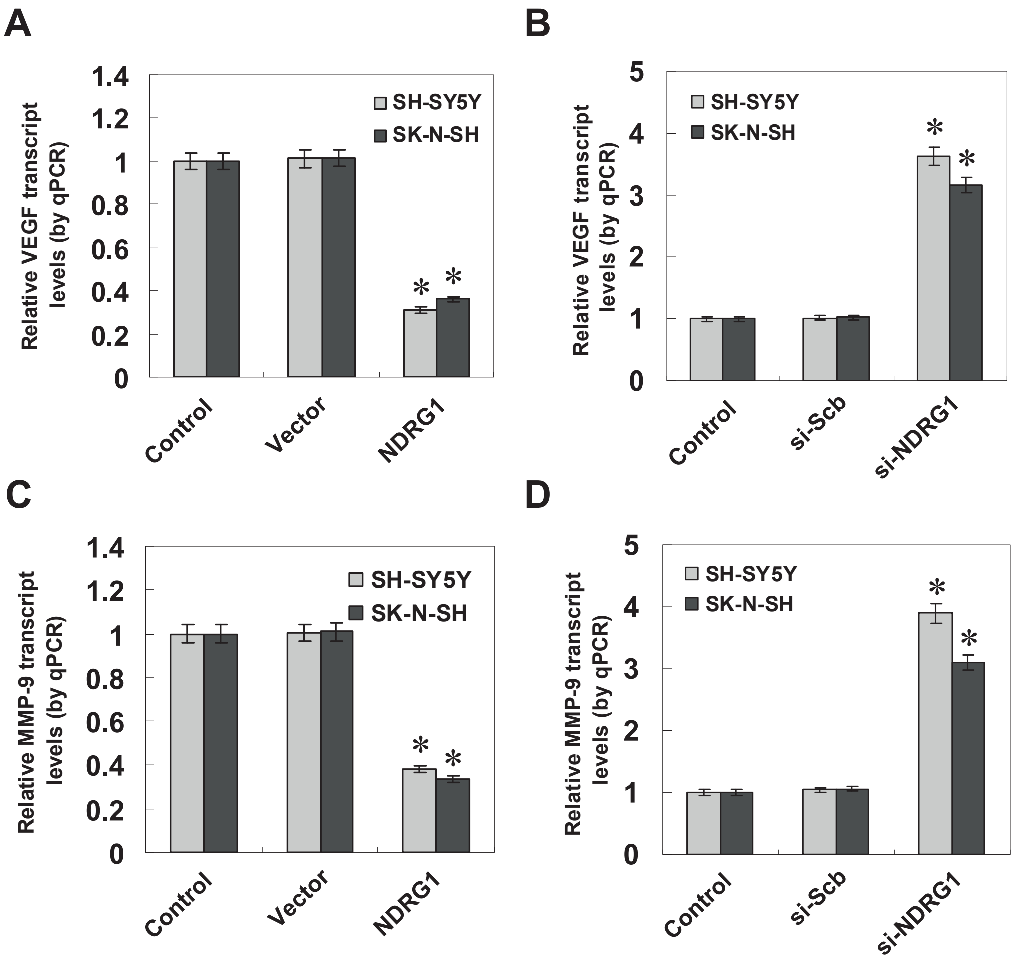

Supplementary Figure S3: Direct regulation of VEGF and MMP-9 by NDRG1 in NB cells. Transfection of NDRG1 construct or si-NDRG1 (100 nmol/L) into SH-SY5Y and SK-N-SH cells altered the expression of VEGF (**A** and **B**) and MMP-9 (**C** and **D**), respectively (* $P < 0.01$ vs. control).

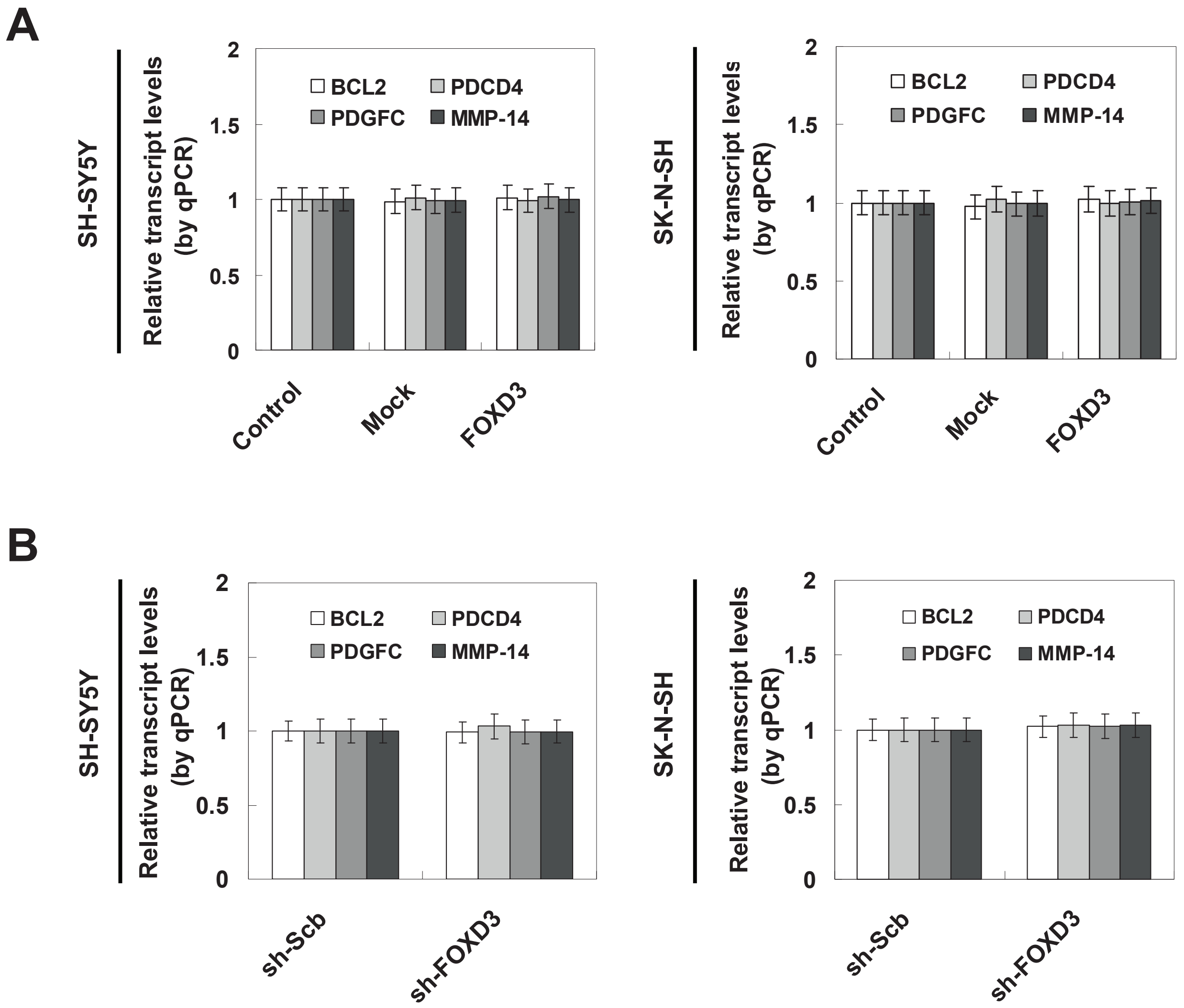

Supplementary Figure S4: FOXD3 did not affect the expression of other potential target genes. Real-time quantitative RT-PCR indicated that stable over-expression (**A**) or knockdown (**B**) of FOXD3 into SH-SY5Y and SK-N-SH cells did not affect the expression of BCL2, PDCD4, PDGFC, or MMP-14.

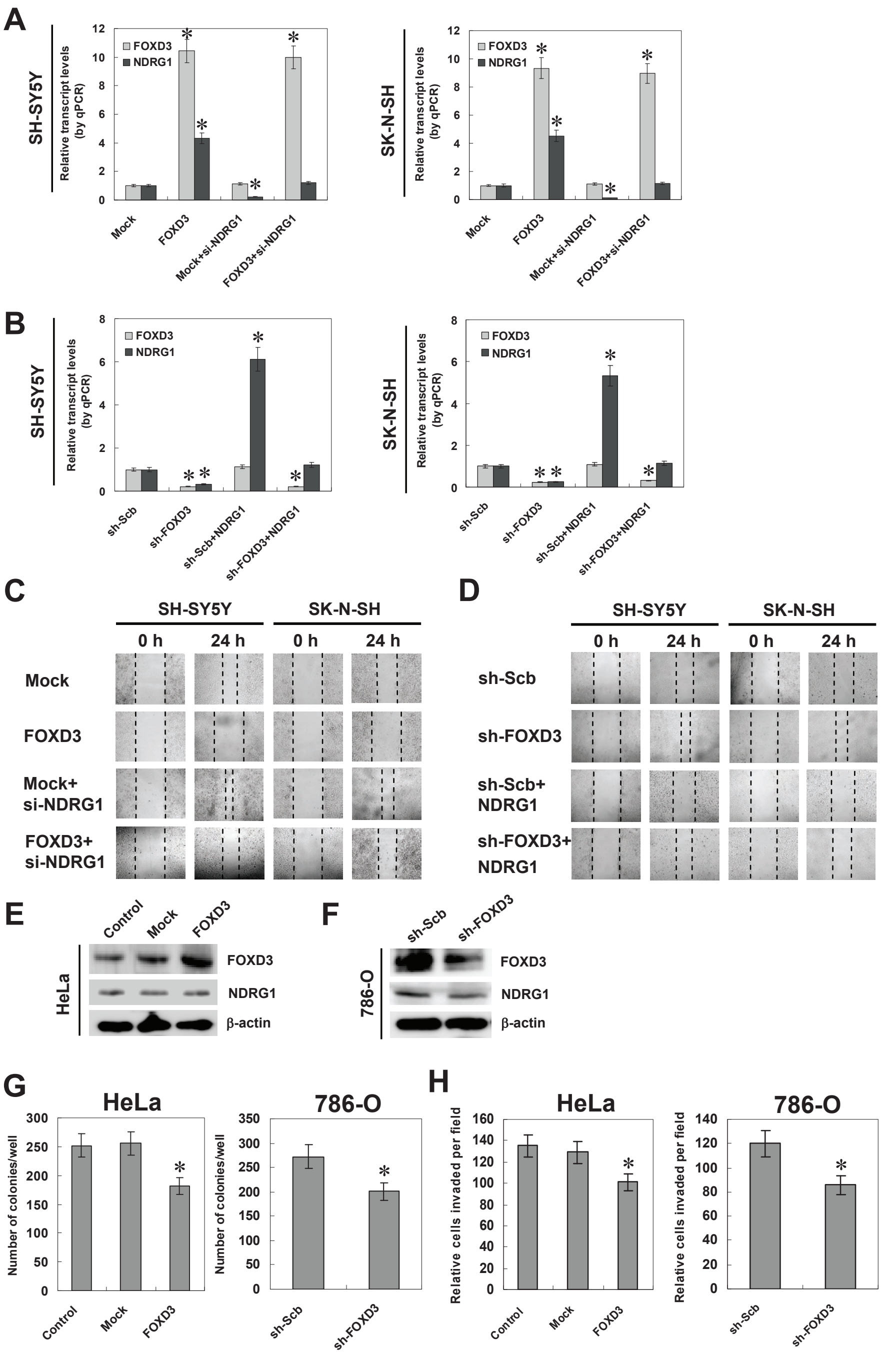

Supplementary Figure S5: Ectopic expression and knockdown of FOXD3 in tumor cells. (**A** and **B**) real-time quantitative RT-PCR indicated that stable transfection of FOXD3 expression vector or short hairpin RNA targeting FOXD3 (sh-FOXD3) vector into SH-SY5Y and SK-N-SH cells resulted in enhanced or decreased transcript levels of FOXD3, when compared to those transfected with empty vetor (mock) and scramble short hairpin RNA (sh-Scb), respectively. Transfection of NDRG1 siRNA (100 nmol/L) or NDRG1 expression vector restored the changes of NDRG1 expression induced by stable FOXD3 over-expression and knockdown, respectively (* $P < 0.01$ vs. mock or sh-Scb). (**C** and **D**) the migration of FOXD3 over-expressing or knocking down SH-SY5Y and SK-N-SH cells was significantly reduced or increased when compared to mock or sh-Scb, which was rescued by transfection of NDRG1 siRNA and NDRG1 vector, respectively. (**E** and **F**) western blot indicated that transfection of FOXD3 expression or sh-FOXD3 vectors resulted in over-expression or knockdown of FOXD3 in cervix cancer cell line HeLa and renal cancer cell line 786-O, without significant changes in NDRG1 expression, when compared to those transfected with mock and sh-Scb, respectively. (**G** and **H**) soft agar and transwell assays indicated that over-expression of FOXD3 attenuated the growth and invasion of HeLa cells, while knockdown of FOXD3 suppressed these biological features of 786-O cells (* $P < 0.01$ vs. mock or sh-Scb).

Supplementary Table S1 FOXD3 expression in human NB tissues

Group	Total number	Expression of FOXD3				Positive rates	P-Value
		−	+	++	+++	(%)	
Age							
< 1 year	20	11	4	3	2	45.0	0.126
≥ 1 year	22	19	2	1	0	13.6	
Differentiation							
Undifferentiated	6	5	1	0	0	16.7	
Poorly differentiated	28	25	1	2	0	10.7	< 0.001
Well differentiated	8	0	4	2	2	100.0	
MKI							
< 200	17	7	4	4	2	58.8	0.003
> 200	25	23	2	0	0	8.0	
INSS stages							
Stage 1-2	14	5	4	3	2	57.1	
Stage 3-4	20	19	1	0	0	5.3	0.018
Stage 4S	8	6	1	1	0	37.5	

FOXD3, forkhead box D3; MKI, mitosis karyorrhexis index; INSS, international neuroblastoma staging system

Supplementary Table S2 Correlation among the expression of FOXD3, NDRG1 and CD31

	FOXD3 expression			
	**Low**	**High**	***R*-value**	***P*-value**
**NDRG1 expression**				
Low	28	1	0.463	0.002
High	8	5		
**CD31 expression**				
Low	6	4	-0.411	0.007
High	30	2		

FOXD3, forkhead box D3; NDRG1, N-myc downstream regulated 1; Pearson's correlation coefficient was applied to determine the expression correlation.

Supplementary Table S3 Primer sets used for qPCR and ChIP

Primer set	Primers	Sequence	Product size (bp)	Application
FOXD3	Forward<br>Reverse	5'-GACGACGGGCTGGAAGAGAA-3'<br>5'-GCCTCCTTGGGCAATGTCA-3'	161	qPCR
NDRG1	Forward<br>Reverse	5'-TGAGGTGAAGCCTTTGGT-3'<br>5'-GAAGAGGGGGTTGTAGCA-3'	199	qPCR
VEGF	Forward<br>Reverse	5'-CTGACGGACAGACAGACAGACAC-3'<br>5'-GCCCAGAAGTTGGACGAAAA-3'	180	qPCR
MMP-9	Forward<br>Reverse	5'-CAGAGATGCGTGGAGAGT-3'<br>5'-TCTTCCGAGTAGTTTTGG-3'	220	qPCR
BCL2	Forward<br>Reverse	5'-TGTGTGGAGAGCGTCAAC-3'<br>5'-ACAGCCAGGAGAAATCAA-3'	175	qPCR
PDCD4	Forward<br>Reverse	5'-AAATGCTGGGACTGAGGA-3'<br>5'-CTTTGGACTGGTTGGCAC-3'	202	qPCR
PDGFC	Forward<br>Reverse	5'-TCCAGCAACAAGGAACAGAACG-3'<br>5'-CAGTACCAGAACCACACCAGCG-3'	289	qPCR
MMP-14	Forward<br>Reverse	5'-GCCTTCTGTTCCTGATAA-3'<br>5'-CCATCCTTCCTCTCGTAG-3'	225	qPCR
β-actin	Forward<br>Reverse	5'-ATCTACGAGGGGTATGCC-3'<br>5'-TAGCTCTTCTCCAGGGAG-3'	227	qPCR
NDRG1(-164/+69)	Forward<br>Reverse	5'-CCCCTACGACTGCTTGCG-3'<br>5'-GGAGCCAGGCGAGGTTTGTTTA-3'	233	ChIP
NDRG1(-34/+110)	Forward<br>Reverse	5'-TGCTGGGACTGCGAGGGTCT-3'<br>5'-CCGAGGGCGGATGGTGAACT-3'	144	ChIP

FOXD3, forkhead box D3; NDRG1, N-myc downstream regulated 1; VEGF, vascular endothelial growth factor; MMP-9, matrix metalloproteinase 9; BCL2, B-cell CLL/lymphoma 2; PDCD4, programmed cell death 4; PDGFC, platelet derived growth factor C; MMP-14, matrix metallopeptidase 14.

Supplementary Table S4 Oligonucleotide sets used for constructs and small interfering RNAs

Oligo Set	Sequences
pEGFP-FOXD3	5'-CGCCCAAGCTTATGACCCTCTCCGGCG-3' (sense);<br>5'-CCGCTCGAGCTATTGCGCCGGCCATTTGG-3' (antisense)
pcDNA3.1-FOXD3	5'-CGCCCAAGCTTATGACCCTCTCCGGCG-3' (sense);<br>5'-CGCGGATCCCTATTGCGCCGGCCATTTGG-3' (antisense)
pcDNA3.1-NDRG1	5'-CCCAAGCTTATGTCTCGGGAGATGCAGGA-3' (sense)<br>5'-CCGCTCGAGCTAGCAGGAGACCTCCATGG-3' (antisense)
pGL3-NDRG1(-759/+69)	5'-GGAAGATCTACGGTGCTAAGGTTGGAAAGGG-3' (sense)<br>5'-CCCAAGCTTGGAGCCAGGCGAGGTTTGTTTA-3' (antisense)
pGL3-NDRG1(-446/+69)	5'-GGAAGATCTCCGAGCTGGTGAGACCTACA-3' (sense)<br>5'-CCCAAGCTTGGAGCCAGGCGAGGTTTGTTTA-3' (antisense)
pGL3-NDRG1(-213/+69)	5'-GGAAGATCTACTGCAGAGCCGACCCACAA-3' (sense)<br>5'-CCCAAGCTTGGAGCCAGGCGAGGTTTGTTTA-3' (antisense)
pGL3-NDRG1(-213/+40)	5'-GGAAGATCTACTGCAGAGCCGACCCACAA-3' (sense)<br>5'-CCCAAGCTTGCGGAGGGCGACTTTATAGGCG-3' (antisense)
pGL3-NDRG1(ΔFOXD3)	5'-GTCGCCCTCCGCCCGGACGTGTTTAAACCTCGCC-3' (sense)<br>5'-ACGTCCGGGCGGAGGGCGACTTTATAGGCG-3' (antisense)
sh-Scb	5'-GCGGACGTTACCGTTAAACAACTCGAGTTGTTTAACGGTAACGTCCGC-3' (sense);<br>5'-GCGGACGTTACCGTTAAACAACTCGAGTTGTTTAACGGTAACGTCCGC-3' (antisense)
sh-FOXD3	5'-AGCATCGAGAACATCATAGGTCTCGAGACCTATGATGTTCTCGATGCT-3' (sense);<br>5'-AGCATCGAGAACATCATAGGTCTCGAGACCTATGATGTTCTCGATGCT-3' (antisense)
si-Scb	5'-GCUUGCUUAUUCGACGUAAtt-3' (sense);<br>5'-UUACGUCGAAUAAGCAAGCtt-3' (antisense)
si-NDRG1	5'-UGGAGUCCUUCAACAGUUUtt-3' (sense);<br>5'-AAACUGUUGAAGGACUCCAtt-3' (antisense)

FOXD3, forkhead box D3; NDRG1, N-myc downstream regulated 1; sh-Scb, scramble short hairpin RNA; si-Scb, scramble small interfering RNA
